# The Impact of COVID-19 on a Large, Canadian Community Emergency Department

**DOI:** 10.5811/westjem.2021.1.50123

**Published:** 2021-05-05

**Authors:** Daniel Dongjoo Lee, Hyejung Jung, Wendy Lou, David Rauchwerger, Lucas B. Chartier, Sameer Masood, Seyon Sathiaseelan, Ahmed Khaled Taher

**Affiliations:** * Temerty Faculty of Medicine, University of Toronto, Toronto, Ontario, Canada; † Dalla Lana School of Public Health, University of Toronto, Toronto, Ontario Canada; ‡ Mackenzie Health, Department of Emergency Medicine, Richmond Hill, Ontario, Canada; § University of Toronto, Department of Medicine, Division of Emergency Medicine, Toronto, Ontario, Canada; ¶ University Health Network, Department of Emergency Medicine, Toronto, Ontario, Canada; || McMaster University, Department of Family Medicine, Hamilton, Ontario, Canada

## Abstract

**Introduction:**

As the COVID-19 pandemic unfolded, emergency departments (EDs) across the world braced for surges in volume and demand. However, many EDs experienced decreased demand even for higher acuity illnesses. In this study we sought to examine the change in utilization at a large Canadian community ED, including changes in patient demographics and presentations, as well as structural and administrative changes made in response to the pandemic.

**Methods:**

This retrospective observational study took place in Ontario, Canada, from March 17–June 30, 2020, during province-wide lockdowns in response to COVID-19. We used a control period of March 17–June 30 in 2018–2019. Differences between observed and expected values were calculated for total visits, Canadian Triage and Acuity Scale (CTAS) groups, and age groups using Fisher’s exact test. Length of stay (LOS), physician initial assessment time (PIA), and top primary and admission diagnoses were also examined.

**Results:**

Patient visits fell to 66.3% of expected volume in the exposure period (20,901 vs 31,525, P<0.0001). CTAS-1 (highest acuity) patient volumes dropped to 86.8% of expected (P = 0.1964) while CTAS-5 (lowest acuity) patient volumes dropped to 32.4% of expected (P <0.0001). Youth (0–17), adult (18–64), and senior (65+) visits all decreased to 37.4%, 71.7%, and 72.9% of expected volumes, respectively (P <0.0001). Median PIA and median ED LOS both decreased (1.1 to 0.6 hours and 3.3 to 3.0 hours, respectively). The most common primary diagnosis in both periods was “other chest pain.” Viral syndromes were more prevalent in the exposure period. The top admission diagnoses were congestive heart failure in the control period (4.8%) and COVID-19 in the study period (3.5%).

**Conclusion:**

ED utilization changed drastically during COVID-19. Our ED responded with wide stakeholder engagement, spatial reorganization, and human resources changes informed by real-time data. Our experiences can help prepare for potential subsequent “waves” of COVID-19 and future pandemics.

## INTRODUCTION

The first case of COVID-19 in Canada was identified in Toronto, Ontario, on January 25, 2020. Over the next few months, every province and territory in Canada declared a state of emergency and instituted lockdowns in response to increasing infection spread, which reached a total of 1,155,834 cases as of April 23, 2021.^1^ Provincial and local health systems prepared for worst-case scenarios as they observed dire situations in areas that were hit hard early including Italy, Spain, and areas in the United States. 2,3

Based on challenging international experiences, many Canadian emergency departments (ED) sought to prepare for a high flux of patients with infectious symptoms.^4^–^6^ However, as the pandemic unfolded, many EDs did not appear to experience surges, but rather decreases in volumes. In the prehospital realm, emergency medical system (EMS) activations for potential life-threatening presentations decreased in the US and Europe.^7^,^8^ Substantial decreases in ED volumes have been noted worldwide, even in countries with high COVID-19 burden.^9^,^10^ Moreover, early studies from Canada and worldwide found decreased visits for heart failure, stroke, and acute myocardial infarctions.^11^–^14^

Given the large role of EDs in the response to the COVID-19 pandemic, it is important to investigate the landscape of ED utilization during the initial period. The objective of this study was to characterize the utilization of a large community hospital in Ontario, the most populous province, after the declaration of province-wide lockdowns, as well as to explore observed local changes in response to these effects. This included changes in incoming patient demographics, common presentations, and internal indicators of operations such as length of stay (LOS) and physician initial assessment (PIA) times.

These insights can help inform stakeholders in the provision of emergency care by examining how populations respond to pandemics in their decisions to visit EDs. The findings have implications for human resource planning, hospital resource management, supply chain manufacturing and procurement, and local continuing education initiatives.

## METHODS

### Study Design and Time Period

This was a retrospective, population-based cohort study conducted in the Mackenzie Health (MH) Hospital ED. The study period was from March 17–June 30 in the years 2018–2020. The exposure time period starts March 17, 2020, during which the provincial government of Ontario declared a state of emergency response to COVID-19 and instituted lockdowns of non-essential services. Data collection ended on June 30, 2020. We used a control period of March 17–June 30 in the years 2018–2019 to obtain baseline characteristics for examined variables. This study was approved by the Southlake Regional Health Centre Research Ethics Board.

**Population Health Research Capsule**

What do we already know about this issue?

*Emerging evidence shows that COVID-19 significantly disrupted demand for healthcare internationally.*

What was the research question?

*How has COVID-19 changed the demand for emergency care in large community hospitals?*

What was the major finding of the study?

*We found decreased volumes, increased acuity, older demographics, and higher proportions of infectious presentations.*

How does this improve population health?

*By understanding the impact of pandemics on population-level demand for emergency care, we can better prepare for future outbreaks.*

### Study Setting

Mackenzie Health is a large 506-bed community hospital in Richmond Hill, Ontario. The ED received 111,384 visits in 2019. Richmond Hill is a city of 195,022 in Ontario, a province with a population of 13,448,494.^15^ The pre-COVID-19 ED was organized into three main zones. The non-ambulatory zone included a five-bed resuscitation room, five mental health beds, 14 acute care rooms (one isolation), and 20 designated hallway stretchers for overflow. It also included a subacute zone with 15 beds, four of which were isolation beds. The ambulatory zone, for patients who did not need a bed but needed thorough evaluation, included 10 assessment rooms and 30 treatment chairs for patients awaiting treatment or for results. Finally, the minor-treatment zone included six assessment rooms, an eye examination room, and a procedure room, the latter mostly for musculoskeletal injuries.

### Data Collection

We retrieved data from the hospital electronic database (Epic Systems Corporation, Verona, WI). We examined total daily visits and acuity level via the Canadian Emergency Department Triage and Acuity Scale (CTAS).^16^ We also examined patient age (youth 0–17; adult 18–64; senior 65+), physician initial assessment (PIA) time, length of stay (LOS) duration, primary (most responsible) diagnosis for the visit, and admission diagnoses.

Primary diagnoses were defined as the most responsible diagnosis coded in the hospital electronic database according to the *International Classification of Diseases, 10th rev* (ICD-10) format. We compared the volume and proportion of the top five primary diagnoses to the 2018–2019 control period. This was also done for the top five admission diagnoses. Finally, the volume and proportion of acute myocardial infarction (AMI) and acute stroke were examined for the purpose of comparison to emerging literature on the impact of COVID-19 on those conditions in other jurisdictions.^11^–^14^ We abstracted AMI visits as visits with the most responsible diagnosis field coded as either ICD-10 I21 or ICD-10 I22,^17^ while acute stroke was abstracted with most responsible diagnosis fields coded as ICD-10 I60, ICD-10 I61, ICD-10 I62, ICD-10 I63, and ICD-10 I64 (Table 1).^18^

### Data Analysis

We compared observed numbers of visits to expected numbers as projected by the method in Johnston et al (2002) (Box A1).^19^ The expected numbers were calculated using the dates March 17–June 30, 2018–2019, and pre-COVID-19 2020 data to account for seasonality as well as year-to-year variation. We conducted the comparisons between observed and expected numbers using Fisher’s two-tailed exact test. The Bonferroni correction (0.05/8 = 0.00625) was used to adjust for multiple testing in subgroup analyses for CTAS and age (Table 2). We summarized LOS and PIA data by interquartile range (IQR), and 90^th^ percentiles. Categorical variables of primary diagnoses and admission diagnoses were summarized by percentages. We performed statistical analysis using SAS version 9.4 software (SAS Institute Inc., Cary, NC).

## RESULTS

### Volumes

**Figure 1:**
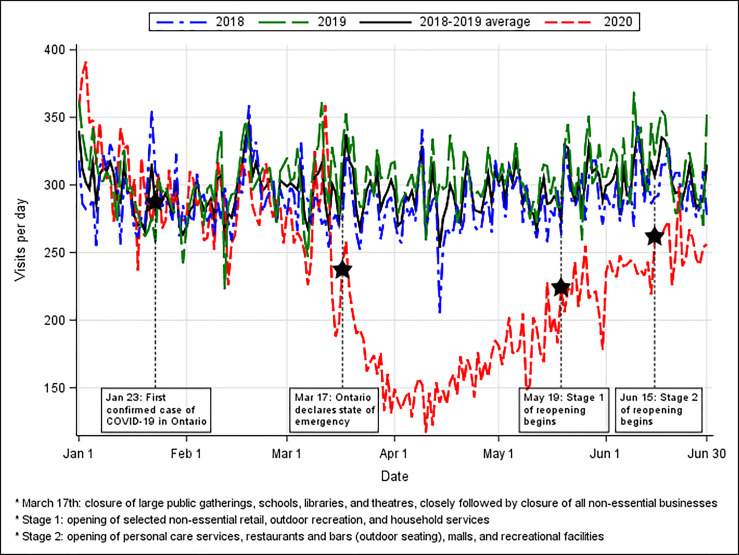
Daily visits to the emergency department from January 1–June 30 for the years 2018 to 2020. *March 17th: closure of large public gatherings, schools, libraries, and theatres, closely followed by closure of all non-essential businesses *Stage 1: opening of selected non-essential retail, outdoor recreation, and household services *Stage 2: opening of personal care services, restaurants and bars (outdoor seating), malls, and recreational facilities

The number of ED visits during 2020 fell starting in mid-March, close to Ontario’s declaration of a province-wide state of emergency on March 17 (Figure 1). The total volume during the exposure period was 66.3% of expected volumes (20,901 vs 31,525, *P*<0.0001) (Table 2).

### Canadian Triage and Acuity Scale

During the exposure period the volume of all CTAS categories fell compared to expected values (32.4%–86.8%) (Table 2). The volume of CTAS-1 (highest acuity) patients experienced the smallest reduction (86.8% of expected, 95% confidence interval [CI], 69.8–107.8) while the volume of CTAS-5 (lowest acuity) patients experienced the greatest reduction (32.4% of expected, 95% CI, 27.9–37.5). Decreases in CTAS 2–4 patients were statistically significant (*P*<0.0001 for all groups).

### Age

All three age groups’ (youth, adult, senior) volumes during the exposure period were below expected (37.4%, 71.7%, and 72.9% of expected, respectively; all *P*-values <0.0001) (Table 2). Youth visits dropped more than adults and seniors compared to expected values. Additionally, the proportion of youth visits fell from 16.6% in 2018–2019 to 9.2% in 2020.

### Length of Stay and Physician Initial Assessment Times

The median ED LOS decreased from 3.3 hours (Q1–Q3: 1.9–5.6) across 2018–2019 to 3.0 hours (Q1–Q3: 1.7–5.1) during the exposure period (Table 3). The 90th percentile LOS fell from 10.7 to 8.8 across the same timeframe. The median time to PIA decreased from 1.1 hours (Q1–Q3: 0.6–1.7) in 2018–2019 to 0.6 hours (Q1–Q3: 0.3–1.0) during the exposure period. The 90th percentile PIA fell from 2.3 hours to 1.5 hours across the same timeframe.

### Primary Diagnoses

The most common primary diagnosis in both the control and exposure periods was *R073 - Other chest pain* (4.1% and 4.9% of all visits, respectively) (Table 4). In the exposure period *U071 – Coronavirus Disease 2019, virus identified* became the third most common primary diagnosis. *B349 – Viral infection, unspecified* and *J069 – Acute URTI, unspecified* entered the most common primary diagnoses as well.

The proportion of AMI and acute strokes both increased (0.1% to 0.4% and 0.6 to 0.8%, respectively). The absolute number of AMIs increased from an average of 45 per year over 2018–2029 to 83 in 2020 (84% increase). The absolute number of acute strokes decreased from an average of 188 in 2018–2019 to 177 in 2020 (5.9% decrease).

### Admissions

Top admission diagnoses are outlined in Table 5. The top cause for admission in 2018–2019 was congestive heart failure (4.8%), whereas the top cause in 2020 was COVID-19 (3.5%). Acute appendicitis, urinary tract infections, and acute renal failure were within the top five causes of admission in both 2018–2019 and 2020.

## DISCUSSION

### Interpretation of Findings

In this study we noted significant decreases in ED volumes, particularly for youth patients (aged 0–17 years) and lower acuity patients (CTAS 4–5). This was accompanied by decreases in PIA and LOS. COVID-19 entered the most common primary diagnoses overall as well as the most common primary diagnoses causing admission. Infectious primary diagnoses became more common as well. We also noted a large increase in AMIs and a slight decrease in acute strokes, although they both increased in terms of the proportion of total ED visits. Additionally, there was a slight decrease in the number of patients who were dead on arrival to the ED during the study periods in 2018 and 2019 to 2020 (45, 44, and 39, respectively).

The decreases in ED volumes are similar to international reports.^10^ The reasons for this phenomenon have not been determined but may be a combination of several theories: 1) patient anxiety surrounding hospitals as a source of contagion;^20^ 2) public health messaging about “flattening the curve” and a fear of exceeding health system capacity; 3) a reduction in risk-related activities such as biking, drinking alcohol outdoors, driving as a result of the population staying at home; and 4) fewer medical procedures and operations from the cancellation of elective procedures to protect personal protective equipment supply and hospital bed capacity. Similarly, significant drops in ED volumes were seen during the 2003 severe acute respiratory syndrome (SARS) outbreaks in Toronto and Taiwan.^21^–^23^ The LOS and PIA times also decreased during this time period presumably due to reduced patient volumes and thus reduced ED workloads.

There were significant drops in low-acuity patients, similar to what was observed in the SARS ourbreaks, 23 which may again reflect hesitancy to visit the hospital for less-severe issues. The proportion of youth visits decreased more than adults and seniors; this was also seen during the SARS pandemic in Toronto.^21^ Thus, seniors made up a larger proportion of our patient visits. While our ED is equipped with a geriatrics nurse team, future efforts to support an increased presence of a geriatric population may include providing professional education on geriatric emergency care, building a physical ED environment that supports safety and independent function, collaborating with community supports for transitions of care, and increasing geriatric nurse or geriatrician access.^24^

The proportion of influenza-related conditions and respiratory tract infections increased during COVID-19, similar to the increase in influenza-like illness volumes seen in the US during the H1N1 outbreak.^25^ This may have been due to increased awareness about contracting COVID-19, which presents with non-specific symptoms including fever and cough. Acute myocardial infarction and stroke made up a higher proportion of visits during the study period, which may reflect the reduction of lower-acuity visits. However, the absolute volume of AMIs increased while the number of strokes decreased slightly. Our experience is in contrast with international reports, which found decreases in AMIs and larger decreases in the number of strokes.^12^–^14^

### Local Response at the Mackenzie Health Emergency Department

As the makeup of emergency visits changed at MH, department leadership instituted a number of structural and human resource changes to meet the newly changing landscape. This undertaking necessitated ongoing wide stakeholder engagement both within the ED and with the hospital administration. In response to the significant decrease of patient volumes, more agile emergency-physician staffing adjustments were made. The changes resulted in almost monthly changes to the ED staffing template in terms of physician hours and zone coverage to meet demand. These adjustments required ongoing real-time data analysis and feedback, as well as constant engagement with the clinicians in the ED.

Although there was a decrease in visits of all triage levels, lowest severity patients visits (CTAS 4–5) decreased the most in both volume and in proportion. At MH, this led to the conversion of the minor treatment zone into an admitted isolation patient outflow area to support the sub-acute zone. Additionally, the sub-acute zone was fitted with high-efficiency particulate air (HEPA) filters and added COVID-19 screening capabilities resulting in the zone in its entirety becoming a positive pressure area. Lower acuity patients were treated in the ambulatory zone, which was equipped with physically distanced chairs. Patient educational material was presented with signage and on media screens to remind patients of proper hand hygiene and physical distancing in the ED. Hallway beds were also removed to further allow for distancing. However, these changes decreased the total number of available acute care beds from 64 to 37 in the ED. Therefore, surge planning was frequently updated in the case of sharp increases in patient visits.

Additionally, MH experienced an increased proportion of patients presenting with respiratory symptoms. To meet this demand, the resuscitation area was converted into an ambulatory isolation area with eight chairs, two beds, and a new HEPA filtration system. Moreover, a dedicated and physically separate COVID-19 assessment centre was opened at MH, which experienced drastic increases in volumes during the study period (Figure A1). This assessment centre was set up for performing COVID-19 reverse transcription polymerase chain reaction tests for ambulatory low-acuity or asymptomatic patients. The centre was set up with eight assessment rooms overseen by a team of two emergency physicians and dedicated nurses. This allowed for rapid turnaround for low-acuity patients, tailored care for the intended visit, minimized transmission between asymptomatic and symptomatic patients, and facilitated ED medical resources to be used towards higher acuity patients in the ED.

An ongoing focus on infection control was maintained throughout this time period and a multitude of infection control changes were undertaken in response to the pandemic. These included updating triage screening questions; installation of HEPA ventilation in COVID-19 areas; increases in cleaning frequency and use of full-spectrum UV disinfection machines; reserving negative pressure rooms for aerosol-generating medical procedures; and the use of a dedicated multidisciplinary intubating team for high-risk intubations.

### Research Implications

Delayed presentations of critical illnesses mean that clinicians and administrators will need to prepare with downstream exacerbations of time-sensitive conditions that would have otherwise been managed earlier. Therefore, continued proactive advocacy and messaging informing the public of the safety of EDs ahead of subsequent “waves” is crucial.

Additionally, emergency care resources can be distributed to meet new demands. Changes in patient demographics, i.e., a shift towards older patients, can be met with increased geriatric support, such as extended geriatric nurse practitioner hours. Likewise, emergency care resources can be shifted to accommodate for increases in influenza-like or infectious presentations, such as increasing patient isolation capacity.

## STRENGTHS AND LIMITATION

The data collection method ensured a complete sample of all patient visits during the exposure period. Additionally, the method for calculating expected values accounted for both seasonal variation and year-to-year variation.

However, the exposure period was subjectively chosen by expert opinion to be based on Provincial Government actions and may not precisely capture the period in which shifts in public sentiment and emergency care demand occurred. Additionally, the determination of presenting diagnoses was abstracted from a hospital database using ICD codes and were not confirmed via chart review. Some of these common primary diagnoses are subject to interpretation, such as *Z038 – Encounter for observation for other suspected diseases and conditions ruled out*, which limits inferences about most common presentations. Finally, the observed trends may have been influenced by changes in local care provision. Anecdotally, some local family physician offices closed, which may have redirected some low-acuity complaints to the ED. However, no local hospitals closed or were designated as “COVID-19 hospitals”.

## CONCLUSION

Emergency department utilization changed significantly during COVID-19. This large Canadian ED experienced lower volumes, decreased proportions of lower-acuity and younger patients, and an increase in viral illness presentations. The experiences of this local ED can help equip ED administrators with structural and process-based changes for potential subsequent “waves” of COVID-19 and future pandemics.

## Supplementary Information



## Figures and Tables

**Table 1:** International Classification of Diseases codes used to abstract most responsible diagnoses.

Diagnosis	ICD-10 code	ICD-10 description
AMI	I21	Acute myocardial infarction
	I22	Subsequent myocardial infarction*
Acute Stroke	I60	Subarachnoid haemorrhage
	I61	Intracerebral haemorrhage
	I62	Other nontraumatic intracranial haemorrhage
	I63	Cerebral infarction
	I64	Stroke, not specified as haemorrhage or infarction

* Includes infarction of any myocardial site, occurring within 4 weeks (28 days) from onset of a previous infarction.

*ICD-10*, International Classification of Diseases, Tenth Revision, Clinical Modification; *AMI*, acute myocardial infarction.

**Table 2:** Number of emergency department visits during study period March 17–June 30, 2018 to 2020.

Groups	Number of visits (n,%)*			% Ratio of observed to expected in 2020		

	2018	2019	2020 (observed)	2020 (expected)**	Observed/expected % (95% CI)	P-value***
Total visits	30,540	32667	20,901	31,525	66.3 (64.7–68.1)	<0.0001
CTAS						
1-Resuscitation	221 (0.7)	526 (1.6)	310 (1.5)	357	86.8 (69.8–107.8)	0.1964
2-Emergent	10,142 (33.2)	11,562 (35.4)	6,846 (32.8)	9,352	73.2 (70.1–76.5)	<0.0001
3-Urgent	13,713 (44.9)	16,009 (49.0)	10,664 (51.0)	16,663	64.0 (61.7–66.4)	<0.0001
4-Semi-urgent	4,832 (15.8)	3,300 (10.1)	2,303 (11.0)	3,382	68.1 (63.1–73.5)	<0.0001
5-Non-urgent	1,612 (5.3)	1,244 (3.8)	742 (3.6)	2,290	32.4 (27.9–37.5)	<0.0001
Age group						
Youth (0–17)	5,057 (16.6)	5,460 (16.7)	1,916 (9.2)	5,123	37.4 (34.8–40.1)	<0.0001
Adult (18–64)	17,452 (57.1)	18,637 (57.1)	13,282 (63.5)	18,524	71.7 (69.3–74.1)	<0.0001
Senior (65+)	8,031 (26.3)	8,570 (26.2)	5,703 (27.3)	7,823	72.9 (69.3–76.7)	<0.0001

* Observed number of visits during study period (3/17–6/30).

** Expected volumes calculated by method of Johnson et al (2002) (Box A1).

*** Null hypothesis being that observed visits:expected visits = 1.

*CTAS*, Canadian Emergency Department Triage and Acuity Scale.

**Table 3:** Emergency department length of stay and physician initial assessment during study period (March17–June/30 for the years 2018 to 2020).

	2018–2019 average*		2020	

	Median (Q1–Q3)	90th percentile	Median (Q1–Q3)	90th percentile
Length of stay	3.3 (1.9 – 5.6)	10.7	3.0 (1.7 – 5.1)	8.8
Physician initial assessment	1.1 (0.6 – 1.7)	2.3	0.6 (0.3 – 1.0)	1.5

* Average was calculated by dividing the sum of each statistic for 2018 and for 2019 by 2.

**Table 4:** Primary diagnoses during study period (March 17–June 30) for the years 2018 to 2020.

Top #	2018–2019 average*			2020		

	Diagnoses	N (2,018/2,019)	%	Diagnoses	N (2,020)	%
#1	R073 - Other chest pain	1,283/1,308	4.1	R073 - Other chest pain	1,022	4.9
#2	R104 - Other and unspecified abdominal pain	623/688	2.1	Z038 - Encounter for observation for other suspected diseases and conditions ruled out	708	3.4
#3	N390 - Urinary tract infection, site not specified	539/529	1.7	U071 - Coronavirus disease 2019, virus identified	474	2.3
#4	A09 - Infectious gastroenteritis and colitis, unspecified	499/503	1.6	B349 - Viral infection, unspecified	446	2.1
#5	R42 - Dizziness and giddiness	432/529	1.5	J069 - Acute URTI, unspecified	328	1.6

	Acute myocardial infarction	26/63	0.1	Acute myocardial infarction	83	0.4
	Acute stroke	173/202	0.6	Acute stroke	177	0.8

* 2018–2019 average calculated as the average of percentages for the years 2018 and 2019.

*URTI*, Upper respiratory tract infection.

**Table 5:** Top admission diagnoses during study period (March 17–June 30) for the years 2018 to 2020.

Top #	2018–2019 average*			2020		

	Diagnoses	N (2018/2019)	%**	Diagnoses	N (2020)	%**
#1	I500 - Congestive heart failure	180/172	4.8	R073 - Other chest pain	1022	4.9
#2	K358 - Unspecified acute appendicitis	117/92	2.8	Z038 - Encounter for observation for other suspected diseases and conditions ruled out	708	3.4
#3	N390 - Urinary tract infection, site not specified	89/79	2.3	U071 - Coronavirus disease 2019, virus identified	474	2.3
#4	N179 - Acute renal failure unspecified	66/98	2.2	B349 - Viral infection, unspecified	446	2.1
#5	J189 - Pneumonia unspecified	70/86	2.1	J069 - Acute URTI, unspecified	328	1.6

	Acute myocardial infarction	14/46	0.8	Acute myocardial infarction	57	1.8
	Stroke	108/133	3.2	Stroke	108	3.5

* 2018–2019 average calculated as the average of percentages for the years 2018 and 2019.

** Percentages were calculated out of the visits with an admission disposition.

*URTI*, Upper respiratory tract infection.
