# Supplementary material for: The Impact of COVID-19 on a Large, Canadian Community Emergency Department
Source: West J Emerg Med. 2021 May 5;22(3):572–9. doi: 10.5811/westjem.2021.1.50123 (PMC8202991; doi:10.5811/westjem.2021.1.50123)
Supplement: Supplementary file 1 [file wjem-22-572-s001.docx]

# **Appendix**

**
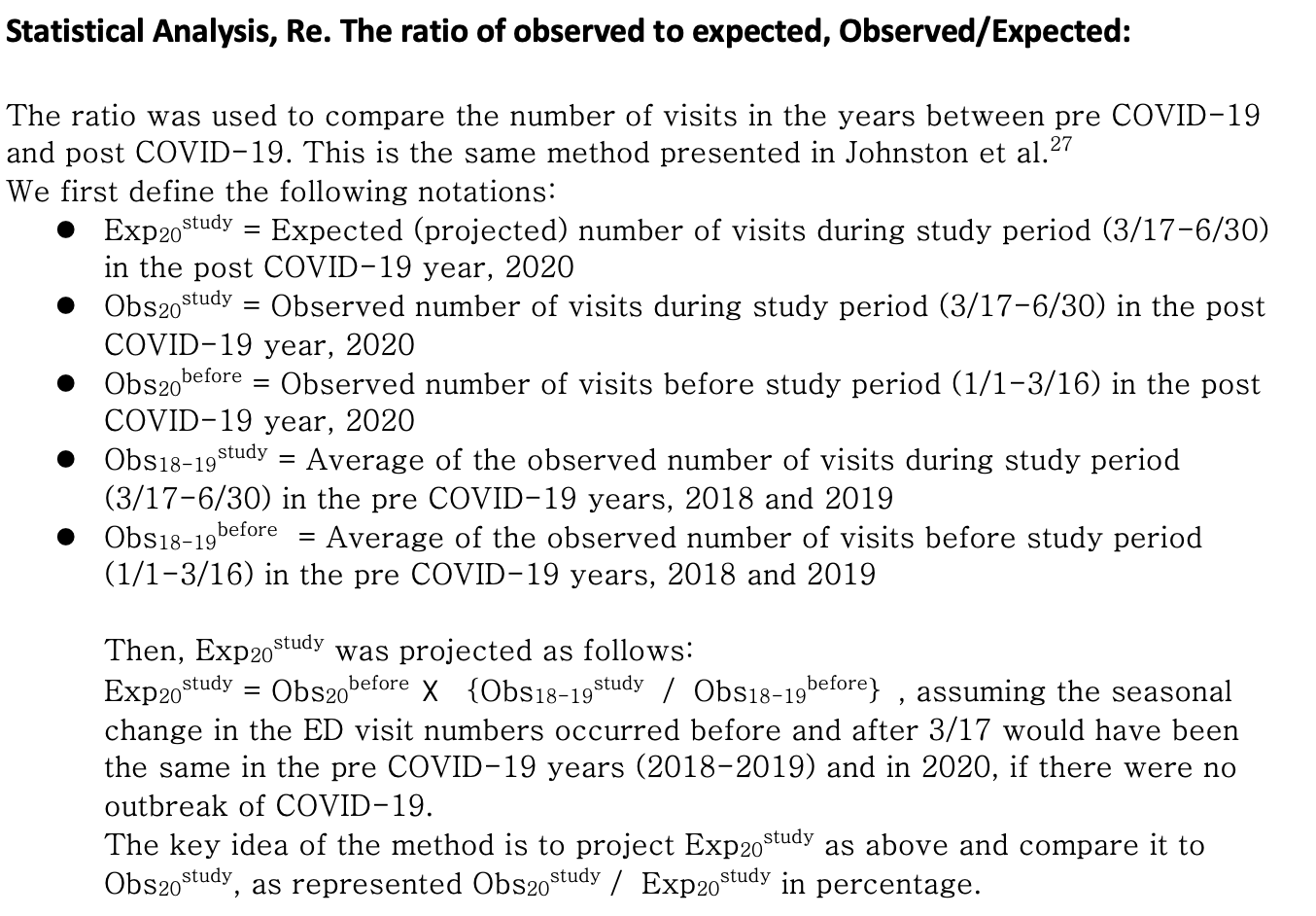
**

**Box A1: Statistical Analysis on the Observed to Expected Ratio.**


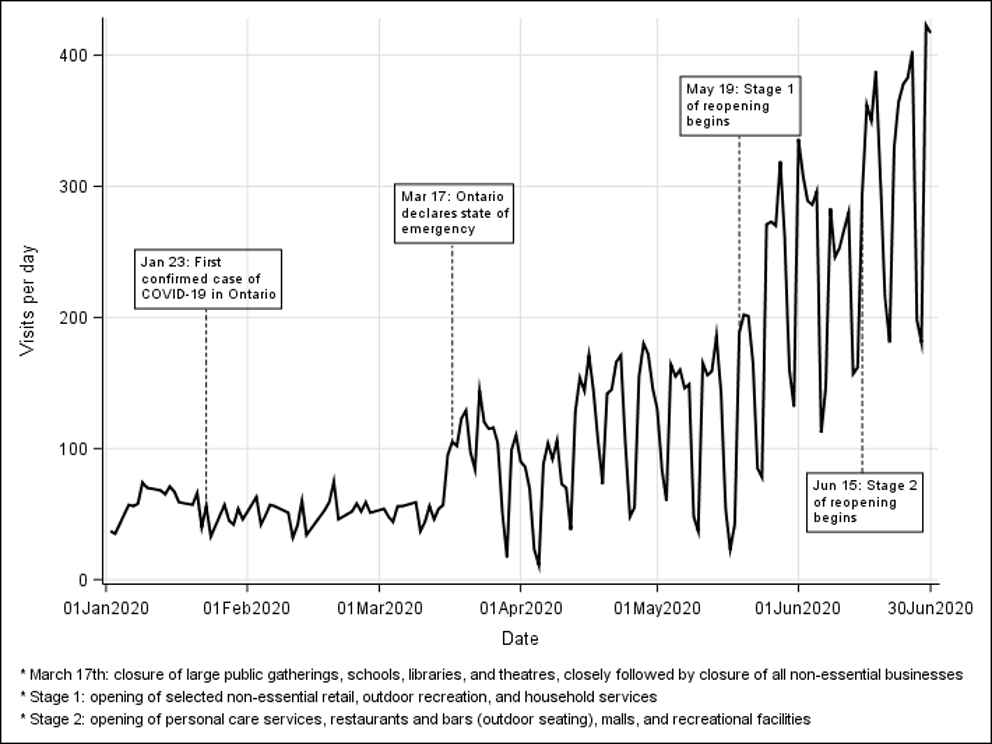


**Figure A1. Daily visits to the COVID-19 Assessment Centre during 6 months from 1/1 to 6/30 for the years 2018 to 2020**.

Note: dips in volumes starting in March correspond with weekends, during which the COVID-19 Centre had reduced hours.

**Table A1. Primary diagnoses during study period (March 17–June 30) for the years 2018 to 2020 (expanded table of Table D)**

| Time | Top 5 Diagnoses | 2018–2019 (Pre-COVID) | | 2020 (Post-COVID) | |
| --- | --- | --- | --- | --- | --- |
|  |  | Top # | % * | Top # | % |
| 2018–2019 (Pre-COVID) | R073 - Other chest pain | 1 | 4.1 | 1 | 4.9 |
| 2018–2019 (Pre-COVID) | R104 - Other and unspecified abdominal pain | 2 | 2.1 | 8 | 1.3 |
| 2018–2019 (Pre-COVID) | N390 - Urinary tract infection site not spec | 3 | 1.7 | 6 | 1.6 |
| 2018–2019 (Pre-COVID) | A099 - Gastroe & colitis of unspec origin | 4 | 1.6 | 18 | 0.9 |
| 2018–2019 (Pre-COVID) | R42 - Dizziness and giddiness | 5 | 1.5 | 15 | 0.9 |
|  |  |  |  |  |  |
| 2020 (Post-COVID) | R073 - Other chest pain | 1 | 4.1 | 1 | 4.9 |
| 2020 (Post-COVID) | Z038 - Obs oth suspected disease & condition | 635 | 0.02 | 2 | 3.4 |
| 2020 (Post-COVID) | U071 - Coronavirus Disease 2019, virus identified | N/A | N/A | 3 | 2.3 |
| 2020 (Post-COVID) | B349 - Viral infection unspecified | 13 | 1.1 | 4 | 2.1 |
| 2020 (Post-COVID) | J069 - Acute URTI unspecified | 26 | 0.7 | 5 | 1.6 |

* Averaged for the years of 2018 and 2019.

**Table A2. Distribution of CTAS during study period by Month**

|  | % 2018-2019 average* | | | | % 2020 | | | |
| --- | --- | --- | --- | --- | --- | --- | --- | --- |
|  | Mar | Apr | May | Jun | Mar | Apr | May | Jun |
| CTAS 1/2 | 37.2 | 38.3 | 35.9 | 31.6 | 30.6 | 36.2 | 35.5 | 33.6 |
| CTAS 3 | 47.4 | 47.4 | 47.3 | 46.1 | 53 | 50.1 | 50.9 | 51.3 |
| CTAS 4/5 | 15.4 | 14.3 | 16.7 | 22.3 | 16.4 | 13.7 | 13.7 | 15.2 |

**CTAS – Canadian Triage and Acuity Scale; CTAS-1 highest acuity to CTAS-5 lowest acuity.**

**Table A3. Distribution of Age during study period by Month**

|  | % 2018-2019 average* | | | | | % 2020 | | | | |
| --- | --- | --- | --- | --- | --- | --- | --- | --- | --- | --- |
|  | Mar | Apr | May | Jun | Mar | | Apr | May | Jun |  |
| Youth (0-17) | 15.6 | 16.5 | 17.5 | 16.4 | 7.1 | | 7.6 | 9.0 | 11.0 |  |
| Adult (18-64) | 57.7 | 56.6 | 56.8 | 57.6 | 68.1 | | 64.6 | 63.7 | 61.1 |  |
| Senior (65+) | 26.7 | 26.9 | 25.7 | 26.0 | 24.8 | | 27.8 | 27.3 | 27.9 |  |
| * 2018-2019 average of percentages for the years 2018 and 2019. | | | | | | | | | | |
| * % were calculated out of total number of visits in each month | | | | | | | | | | |
